# Higher Levels of Postnatal Depressive Symptomatology, Post-Traumatic Growth, and Life Satisfaction among Gay Fathers through Surrogacy in Comparison to Heterosexual Fathers: A Study in Israel in Times of COVID-19

**DOI:** 10.3390/ijerph19137946

**Published:** 2022-06-28

**Authors:** Geva Shenkman, Sigal Levy, Ziv Ben-Dor Winkler, Doriel Bass, Shulamit Geller

**Affiliations:** 1 Baruch Ivcher School of Psychology, Reichman University (IDC Herzliya), Herzliya 4610101, Israel; 2 Statistical Education Unit, The Academic College of Tel Aviv—Yaffo, Yaffo 61083, Israel; levy@mta.ac.il; 3 School of Behavioral Sciences, The Academic College of Tel Aviv—Yaffo, Yaffo 61083, Israel; zivben0@gmail.com (Z.B.-D.W.); bassdoriel@gmail.com (D.B.); shulamit@mta.ac.il (S.G.)

**Keywords:** postnatal depression, growth, gay men, same-sex parents, gay fathers, surrogacy, Israel

## Abstract

This study aimed to explore the psychological welfare, as indicated by postnatal depressive symptomatology, life satisfaction, and posttraumatic growth (growth after contending with stressful birth events), of Israeli gay fathers through surrogacy in comparison to heterosexual fathers. For that purpose, a sample of 167 Israeli fathers (*M* = 35.6, *SD* = 4.4) was recruited (68 identified as gay fathers through surrogacy and 99 as heterosexual fathers). Participants completed questionnaires assessing their postnatal depressive symptomatology, life satisfaction, and sense of posttraumatic growth after becoming fathers. Results indicated that gay fathers through surrogacy reported higher levels of life satisfaction and posttraumatic growth than heterosexual fathers. Yet, gay fathers also reported higher levels of postnatal depressive symptomatology than heterosexual fathers when life satisfaction or posttraumatic growth values were low or medium. The findings were interpreted in light of the hardships associated with cross-border surrogacy and the psychological outcomes associated with succeeding to become fathers after contending with them. The study contributes to the limited literature on postnatal depressive symptomatology and posttraumatic growth among gay fathers through surrogacy and provides clinicians and policymakers with relevant information on the psychological strengths and potential difficulties associated with cross-border surrogacy among gay fathers.

## 1. Introduction

Common routes to gay fatherhood include gay fathers through a previous heterosexual relationship, gay fathers through adoption, gay fathers through shared parenting in agreement with a woman, and gay fathers through surrogacy [1]. Due to developments in fertility technology alongside social and political advancements, gay men are today becoming fathers through surrogacy more than ever before [2,3]. Correspondingly, in recent years, the well-being of gay fathers through surrogacy has gained increased research attention [4,5,6,7]. This research attention adds new information to the relatively limited research on the experiences of men (both heterosexual and gay) around childbirth [8]. However, research exploring indicators of postnatal depressive symptomatology or posttraumatic growth, namely growth after contending with stressful birth events, among this population is scant, and the current study looks to fill this knowledge gap.

We term this study’s targeted outcome as psychological welfare–an overarching term which integrates the philosophies of hedonic and eudaimonic well-being. Hedonic well-being mainly refers to the experience of positive emotional states and life satisfaction, while eudaimonic well-being mainly refers to the presence of meaning and growth [9]. Both types of well-being are often intertwined in the larger mental well-being context which often incorporates negative affective indicators such as depression e.g., [10,11].

Hedonic and eudaimonic well-being have been studied in the context of the transition to parenthood. Findings among heterosexual individuals have shown this transition to be accompanied by decreased levels of hedonic well-being and increased levels of eudaimonic well-being [12]. This pattern is usually explained by both an increase in a sense of personal growth (eudaimonic well-being) when embarking on parenthood and more immediate difficulties affecting life satisfaction (hedonic well-being) such as lack of sleep, growing ambiguity regarding child care, and more marital conflicts over task division e.g., [13]. Postnatal depression has also gained much scholarly attention, first among heterosexual women [14] and, more recently, also among heterosexual men [15], showing a prevalence of 17.7% among the former and 8.8% among the latter [16,17]. Some have suggested that postnatal depressive symptomatology should be considered not only in the first few months after birth but also over longer time periods of two or even three years after birth e.g., [18,19]. Accordingly, the current study addresses postnatal depressive symptomatology among fathers to children aged 0–36 months.

The mentioned pattern of increased eudaimonic alongside decreased hedonic well-being has not been found among gay fathers. Rather, repeated findings in Israel have suggested that fatherhood among gay men is associated with both an increase in meaning in life and an increase in life satisfaction and general happiness [6,20,21]. These findings have usually been explained in the context of the Israeli pronatalist and familistic sociocultural atmosphere. In this context, realizing fatherhood desires, especially after contending with the numerous hurdles restricting gay men’s access to fatherhood, could lead to a sense of victory and elevated levels of of both hedonic and eudaimonic well-being [22,23]. Similarly, as parenthood is considered a primary path to social acceptance and inclusion in Israel’s familistic society, it has been suggested that the transition to fatherhood among gay men may tap into a stronger sense of social acceptance and belongingness that could also explain the elevated levels of well-being [24].

Research regarding depressive symptoms among gay fathers has not tended to detect differences between gay and heterosexual fathers [6,25]. This suggests that, in contrast to the numerous findings showing that childless gay men tend to report more depressive symptomatology than childless heterosexual men e.g., [26,27], fatherhood among gay men is a potential protective factor in mental health [7]. Such affective vulnerability among childless sexual minorities is usually attributed to minority stress, namely, the chronic psychological stress induced by the pressures of stigma, bias, and discrimination [28].

Studies assessing depression among gay fathers have generally used common general depression scales, such as the Center for Epidemiological Studies Depression Scale e.g., [6,11,29] or the Patient Health Questionnaire [30,31]. Only a few studies have focused specifically on postnatal depressive symptomatology among gay fathers [25]. In Israel, where the current study was conducted, there haven’t been reported, to the best of our knowledge, studies that assessed postnatal depressive symptomatology among gay fathers. Particular attention is needed when focusing on the postnatal period or fathering very young children [32], as some of the features of depression identified in these general depression scales, such as changes in appetite and sleep dysregulation, are common and normal features of postpartum adaptation and parenting young children. The Edinburgh Postnatal Depression Scale (EPDS) [33] largely avoids these biological symptoms of depression and was therefore the measure chosen for the current study.

Psychological growth among gay fathers has commonly been assessed via general meaning in life indicators retrieved from a larger context of psychological well-being [21,34]. Growth among gay fathers was suggested to relate with the hardships associated with perusing cross-border surrogacy and the psychological outcomes associated with succeeding to become fathers after contending with them [6]. There therefore remains a need to measure growth among gay fathers who used surrogacy while taking into consideration the stressful experiences accompanying this path. Among new mothers assessing posttraumatic growth, namely growth after contending with stressful birth events such as giving birth to premature babies, has repeatedly pointed to posttraumatic stress symptoms reported during stressful pregnancies as a main predictor of their posttraumatic growth e.g., [35,36,37,38].

### 1.1. The Israeli Sociocultural Context

A guiding theoretical framework for the current study was Cox and Paley’s Family Systems Theory [39], which suggests that individual development and adaptation is shaped by the broader sociocultural context. The sociocultural setting of Israel provides a unique terrain for studying the psychological welfare of gay fathers through surrogacy. On the one hand, Israel is considered a familistic and pronatalist society [40], as demonstrated by Israel having the highest fertility rates of all OECD countries [41]. It has been proposed that Israeli openness to the use of fertility technology, the biblical commandment to be “fruitful and multiply,” and the trauma of the Holocaust, all contribute to these high fertility rates [42]. On the other hand, Israeli legislation restricts sexual minority individuals, especially gay men, who want to become parents. Until recently (January 2022), surrogacy services were illegal for gay couples in Israel, despite being legal for heterosexual couples and single women [43]. Thus, gay men who wished to become fathers through surrogacy had to turn to overseas surrogacy services, usually in the United States [44]. Moreover, LGBTQ+ adoption opportunities are extremely curtailed in Israel [45]. Furthermore, Orthodox Jewish law disapproves and stigmatizes gay individuals, thus creating a sociocultural atmosphere that is often perceived as hostile to sexual minorities [46,47]. This atmosphere may account for the greater anticipation of stigma upon parenthood among Israeli LGBTQ+ individuals than among their heterosexual counterparts [48].

Social context can affect the psychological welfare of parents, and it should thus be noted that our recruitment took place during the second and third waves of the COVID-19 pandemic and its related restrictions, including lockdowns, in Israel. The accumulated stress deriving from health and financial insecurities and from spending more time than usual with children due to social distancing and lockdowns makes any exploration of both postnatal depressive symptomatology and posttraumatic growth among parents with young children extremely relevant [49].

### 1.2. Research Hypotheses

Consistent with the literature discussed above regarding higher levels of hedonic and eudaimonic well-being among gay fathers through surrogacy in comparison to heterosexual fathers and posttraumatic growth among mothers contending with stressful birth events, we hypothesized that gay fathers through surrogacy will report greater life satisfaction and posttraumatic growth than heterosexual fathers. As most studies have not detected differences in depressive symptomatology between gay and heterosexual fathers e.g., [7,11] and there is a lack of data on postnatal depressive symptomatology among gay fathers [25], we did not consolidate a specific hypothesis regarding a difference in postnatal depressive symptomatology between the two groups.

## 2. Materials and Methods

### 2.1. Participants

A total of 197 participants completed the study’s questionnaires; of these, 12 were excluded for not completing the entire survey. Participants who were not fathers, had not become fathers through surrogacy, or did not identify themselves as exclusively gay or exclusively heterosexual were also excluded from the current analysis. Our final sample thus comprised 167 Israeli gay and heterosexual fathers who completed the entire questionnaire set between the ages of 23 and 47 years (*M* = 35.6, *SD* = 4.4). Regarding sexual orientation, 68 identified as gay men who became fathers through surrogacy (aged 29–47; *M* = 37.2, *SD* = 4.2) and 99 identified as heterosexual fathers (aged 23–47; *M* = 34.5, *SD* = 4.2). As shown in Table 1, most participants were born in Israel (93%), had an academic (or partial academic) education (89%), and reported having above average income (70%). The mean number of children was 1.6 and 1.7 in the gay and heterosexual group, respectively. Most participants (98%) reported being in a romantic relationship.

### 2.2. Measures

#### 2.2.1. Demographics

An assessment of sexual orientation was made on a 7-point self-rating scale [50] ranging from 0 (exclusively heterosexual) to 6 (exclusively homosexual). This measure confirmed that participants identified themselves as either exclusively gay or heterosexual men. We also collected other self-rated sociodemographic queries, such as age, country of birth, education level, number of children, romantic relationship status, economic status, and age of the youngest child.

#### 2.2.2. Edinburgh Postnatal Depression Scale (EPDS)

This self-report scale in its Hebrew validation [33,51] consists of 10 items rated on a 4-point severity scale addressing both depressive symptoms (e.g., sadness, self-blame) and anxiety symptoms (e.g., feeling worried, scared, or panicky). A total score is calculated from the 10 items and is used to evaluate postnatal depression, with higher scores reflecting greater symptom severity. Although originally developed as a screening scale for new mothers, it has been found to be a reliable and valid measure of mood in new fathers as well [32,52]. Internal consistency of the EPDS in the present study was good, with McDonald’s Omega = 0.85.

#### 2.2.3. Satisfaction with Life Scale (SWLS)

This scale [53] assesses life satisfaction as the cognitive concomitant of subjective well-being. It consists of five items referring to judgments of one’s life (e.g., “The conditions of my life are excellent”) and was rated by respondents on a scale ranging from 1 (strongly disagree) to 7 (strongly agree). The score is the items’ mean rating. The McDonald’s Omega coefficient of SWLS in the current sample was 0.78. This measure proved to have highly favorable psychometric properties and has been widely used with Israeli samples of gay men, e.g., [54,55].

#### 2.2.4. Post-Traumatic Growth Inventory (PTGI-SF)

This scale [56] was used to measure personal growth and adjustment among fathers following the transition to parenthood [37]. The inventory consists of 10 items that relate to changes on personal, interpersonal, and philosophical levels (e.g., “I found I am stronger than I previously believed”). Participants rated the degree to which the various changes had occurred since the birth of their child on a scale from 0 (not at all) to 5 (very much). This study used the Hebrew translations of the items from the existing 21-item PTGI [57]. A growth score was calculated for each participant as the mean of their responses to all items, with higher scores indicating greater personal growth. The McDonald’s Omega coefficient in the present study was 0.84.

### 2.3. Procedure

After obtaining the Institutional Ethics Committee approval (Protocol 2020153 21.6.2020), participants were recruited using a snowball sampling technique through various social media networks such as Facebook, which focused on fatherhood (heterosexual or gay). Questionnaires were administered from July 2020 to June 2021 through the Qualtrics online platform (www.qualtrics.com). Participants were invited to take part in a survey dealing with fatherhood. They were informed that the questionnaires were anonymous and that participation was completely voluntary. All participants signed an informed consent form. Participants were told they could abandon the questionnaires and withdraw from the study at any point. They were given the researchers’ contact details and debriefed following the completion of the questionnaires.

### 2.4. Data Analysis

Statistical analyses were conducted in SPSS (version 25). Pearson, point biserial, and Cramer’s *V* coefficients examined the correlations between the sociodemographic and study variables. Multivariate analysis of variance (MANOVA) tested the effect of sexual orientation on psychological welfare (postnatal depressive symptomatology, life satisfaction, and posttraumatic growth). A power analysis was conducted by G*Power 3.1.9.6 software [58] in order to determine the required sample size. This analysis indicated that the current sample size guaranteed close to 100% power for detecting a moderate size effect at a 5% significance level for the study hypothesis using a MANOVA analysis with two groups, one independent variable, and three dependent variables. Marginally significant results (0.05 < *p* < 0.09) were presented following Altman’s [59] suggestion to present such results when they appear clinically important. We have also used the PROCESS computation tool for SPSS (Model 1) [60] to explore simple slope analysis [61] and to examine more closely the interaction effect between sexual orientation and posttraumatic growth when predicting postnatal depressive symptomatology and the interaction effect between sexual orientation and life satisfaction when predicting postnatal depressive symptomatology.

## 3. Results

Correlations between the sociodemographic and the study variables showed that none of the former significantly correlated with any of the outcome measures. They therefore were not included as covariates in the model. Also, due to a technical error, sociodemographic measures were not recorded for about 50 participants. This also strengthened the rationale not to control for sociodemographic variables in order not to reduce statistical power. Associations between the study main variables did find a negative correlation between postnatal depressive symptomatology and life satisfaction (r = −0.46, *p* < 0.001).

### 3.1. Group Differences

Group differences were tested using a MANOVA analysis, with psychological welfare indicators, namely, postnatal depressive symptomatology (EPDS), growth (PTGI), and life satisfaction (SWLS), as outcome variables. The overall group effect was significant, *F* (3159) = 8.3, *p* < 0.001, partial eta-squared = 0.14. Univariate analysis, presented in Table 1, showed that gay fathers had higher levels of both postnatal depressive symptomatology and posttraumatic growth than heterosexual fathers. Gay fathers also had higher levels of life satisfaction than heterosexual fathers, yet this effect was marginal (*p* = 0.053).

We also ran two exploratory moderation models: one with posttraumatic growth as a moderator, and one with life satisfaction. This was an attempt to better understand the results showing greater postnatal depressive symptomatology among gay fathers in comparison to heterosexual fathers, as they appear to be new and not in line with prior findings that did not detect differences in general depressive symptomatology between gay and heterosexual fathers.

### 3.2. Moderating the Association between Sexual Orientation and Postnatal Depressive Symptomatology by Posttraumatic Growth

**Figure 1:**
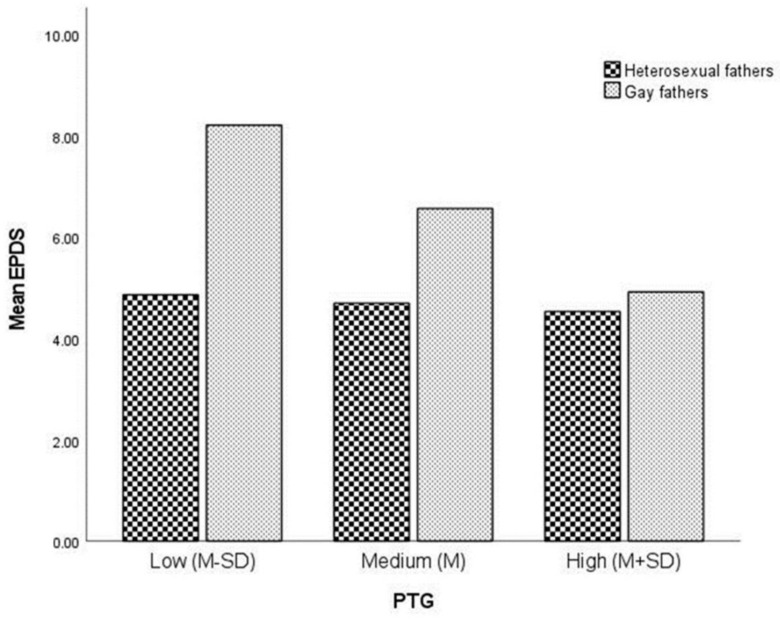
Group by posttraumatic growth (PTGI) interaction affecting Edinburgh Postnatal Depression Scale (EPDS). Simple effects were evaluated at PTGI = 2.80 (low), 3.70 (medium), and 4.60 (high).

To explore the moderating effect of posttraumatic growth on group differences in postnatal depressive symptomatology, we used the PROCESS commutation tool (model 1). The results of this analysis are presented in Table 2. The significant interaction effect of sexual orientation and posttraumatic growth on postnatal depressive symptomatology increased the explained variance by 2.3%, as demonstrated in Figure 1. Simple effects were estimated at low (*M* − *SD*), medium (*M*) and high (*M* + *SD*) values of posttraumatic growth (PTGI). The results of this analysis showed that gay fathers had higher levels of postnatal depressive symptomatology than heterosexual fathers only when posttraumatic growth values were low or medium. These results are shown in Table 3. The results also suggest that the effect of posttraumatic growth on postnatal depressive symptomatology is stronger for gay fathers than for heterosexual fathers.

### 3.3. Moderating the Association between Sexual Orientation and Postnatal Depressive Symptomatology by Life Satisfaction

**Figure 2:**
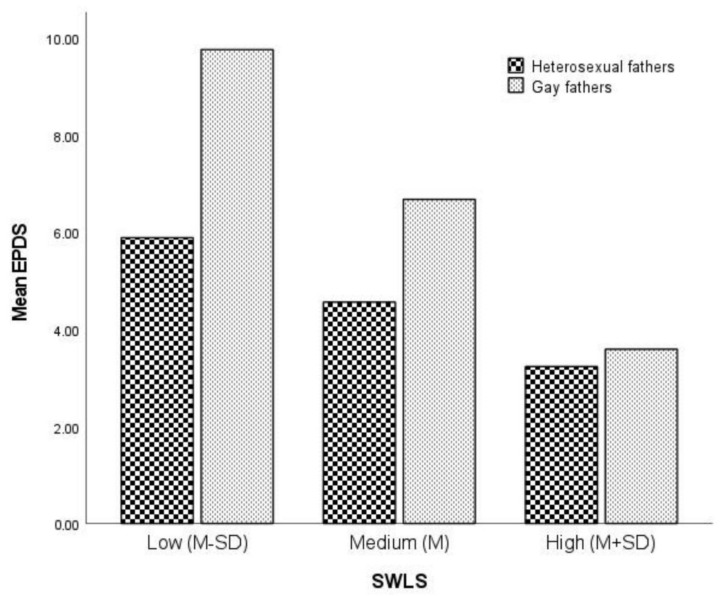
Group by satisfaction with life (SWLS) interaction affecting Edinburgh Postnatal Depression Scale (EPDS). Simple effects were evaluated at SWLS = 4.30 (low), 5.30 (medium), and 6.20 (high).

To explore the moderating effect of life satisfaction on group differences in postnatal depressive symptomatology, we used the PROCESS commutation tool (model 1). The results of this analysis are presented in Table 4. The significant interaction effect of sexual orientation and life satisfaction on postnatal depressive symptomatology increased the explained variance by 4.0%, as demonstrated in Figure 2. Simple effects were estimated at low (*M* − *SD*), medium (*M*), and high (*M* + *SD*) values of life satisfaction (SWLS). The results of this analysis showed that gay fathers had higher levels of postnatal depressive symptomatology than heterosexual fathers only when life satisfaction values were low or medium. These results are shown in Table 5. The results also suggest that the effect of life satisfaction on postnatal depressive symptomatology is stronger for gay fathers than for heterosexual fathers.

## 4. Discussion

In line with our hypotheses, gay fathers through surrogacy reported higher levels of life satisfaction and posttraumatic growth than heterosexual fathers (the difference in life satisfaction was marginal). Although we did not expect a difference in postnatal depressive symptomatology between gay and heterosexual fathers, the former did report higher levels of postnatal depressive symptomatology than the latter. Further analyses specified that gay fathers had higher levels of postnatal depressive symptomatology than heterosexual fathers only when life satisfaction or posttraumatic growth values were low or medium.

The findings displaying higher levels of hedonic (life satisfaction) and eudaimonic (growth) well-being among gay fathers through surrogacy in comparison to heterosexual fathers are in line with prior findings [6,20,21]. However, the novelty of the current findings is that sense of growth was assessed for the first time among gay fathers by relating to the possible stress associated with complex birth circumstances. More specifically, gay fathers through surrogacy reported higher levels of posttraumatic growth than heterosexual fathers when addressing their sense of growth following the transition to parenthood. Previous findings among mothers revealed that contending with stressful birth circumstances, such as giving birth to premature babies or having difficulty getting pregnant, was associated with a greater sense of growth as indicated by posttraumatic growth e.g., [36,38]. As mentioned before, in Israel, where, until recently, gay men were not allowed to use surrogacy services within the country’s borders, gay men who wished to become fathers through surrogacy had to turn to overseas surrogacy services, usually in the United States [44]. This journey to fatherhood often incorporates dealing with stressful circumstances, such as concerns deriving from the geographic distance from the pregnant woman [62] and the many financial, legal, and bureaucratic hardships involved in cross-border surrogacy [63]. These stressful circumstances might promote adaptation that manifests itself in a heightened sense of growth, such as was found among women contending with difficulties in their journey to motherhood e.g., [35,37].

Moreover, it has been suggested that the sense of victory on succeeding to become gay fathers in the Israeli pronatalist sociocultural context, which highly cherishes childrearing and yet imposes restrictions on gay men who want to become fathers, is another possible contributor to the higher levels of life satisfaction and growth found among Israeli gay fathers [22].

Our results revealed that gay fathers through surrogacy reported more postnatal depressive symptomatology than heterosexual fathers. This does not conform with previous findings which detected no differences in depressive symptomatology between gay and heterosexual fathers [7,11,25]. An explanation for our results could be the time period of data collection. Data collection took place during the second and third waves of COVID-19 and its related restrictions, including social isolations and lockdowns, in Israel. Although the stress derived from both health and financial insecurities and spending more time than usual with partners and children was probably evident for both gay and heterosexual parents, it is possible that these added pandemic stressors alongside minority stress [28] reached a threshold that manifested in more depressive symptomatology among gay fathers. This notion of cumulative stress among sexual minorities is in line with prior findings showing that among gay men, mental health vulnerability might be exposed when additional threats are realized: for example, a combination of early family-based vulnerability with the difficulties relating to minority stress might lead to an environment which is conducive to more depressive symptoms [64].

As the results regarding the differences in postnatal depressive symptomatology between the groups were novel and did not conform with previous literature [7,25], we further explored the moderating role of life satisfaction and posttraumatic growth on these differences. We found that gay fathers had higher levels of postnatal depressive symptomatology than heterosexual fathers only when life satisfaction or posttraumatic growth values were low or medium. These results also suggest that the effects of posttraumatic growth and life satisfaction on postnatal depressive symptomatology were stronger for gay fathers than for heterosexual fathers. This highlights the protective role of both hedonic (life satisfaction) and eudaimonic (growth) well-being on the affective vulnerability of postnatal depressive symptomatology among gay fathers. These results are in line with Shmotkin’s model on the pursuit of happiness in the face of adversity [65]. This model suggests that both subjective well-being, commonly indicated by life satisfaction, and meaning in life, commonly indicated by measures of purpose and growth, can regulate and even reconstruct hostile world scenarios in order to maintain a positive psychological environment [66]. Implementation of this model on the current findings could suggest that when life satisfaction or sense of growth are not accessible enough for gay fathers through surrogacy to regulate or reconstruct current adversities, an affective vulnerability of postnatal depressive symptomatology might become apparent.

### Strengths and Limitations

A main strength of the current study is its exploration of the psychological welfare of gay fathers through cross-border surrogacy via indicators that have rarely been examined among this population: postnatal depressive symptomatology and posttraumatic growth relating to becoming fathers. It thus contributes novel information to the literature about the welfare of gay fathers through surrogacy. Moreover, the study’s discovery of the moderating role of life satisfaction and posttraumatic growth on the association between sexual orientation and depressive symptomatology among fathers expands Shmotkin’s model of happiness in the face of adversity [65] to the population of gay fathers.

Alongside these strengths, some limitations should be considered. First, the study relied solely on self-reports and is thus was vulnerable to self-presentation biases. Second, the collected sample was not based on a random or representative sample. Third, the correlational research design did not allow for causal interpretations. Fourth, sociodemographic measures were not recorded for about 50 participants, which restricted the ability to control for sociodemographics between the two study groups. Fifth, we did not have information about the quality of fathers’ relationships or children’s developmental problems, which might be related to the dependent variables and thus should be monitored when comparing the study groups. Sixth, studying psychological welfare issues in the first and second waves of COVID-19 might have produced results that should be very cautiously generalized to less acute circumstances. Lastly, it is also unclear whether a small number of participants’ partners completed this survey, thus introducing an in-accountable level of dependency within the data. Future research should ensure the monitoring of this variable.

## 5. Conclusions

The current study found that levels of life satisfaction, sense of posttraumatic growth, and postnatal depressive symptomatology were higher among gay fathers through surrogacy than heterosexual fathers. A more nuanced examination showed that differences in postnatal depressive symptomatology between the groups were apparent only when levels of life satisfaction and posttraumatic growth were low or moderate. Future studies should further explore postnatal depressive symptomatology among other sexual minority parents, preferably in less challenging circumstances than COVID-19 restrictions and lockdowns. As the current comparative approach to gay and heterosexual counterparts has been criticized for focusing primarily on differences based on sexual identities and disregarding other identities that are prominent to the experience of LGBTQ+ individuals and families [67], we recommend that future studies explore, among LGBTQ+ parents, more variables that can mitigate postnatal depressive symptomatology, such as personality features, positive and negative emotions, satisfaction with romantic relationship, awareness of minority stress, and the age of the youngest child.

Policymakers should pay attention to the current results which may relate to the legal climate in Israel that, until recently, banned gay men from accessing surrogacy services in Israel. Such institutional discrimination seems to adversely affect the well-being of sexual minorities [68,69]. Our findings are also relevant to mental health professionals working with sexual minorities. Although most of the EPDS scores were below the common clinical cutoff (>12) for postnatal depression [19], the findings still capture subclinical affective vulnerability, which professionals should be aware of. Attention to this may promote more sensitive interventions with sexual minority clients.

## Figures and Tables

**Table 1:** Group comparisons in demographic variables and in the main study variables.

Sociodemographic Variables	Gay Fathers (N = 47)		Heterosexual Fathers (N = 70)		F/Chi-Square	*p*
	*M* (*SD*), Range	N (%)	*M* (*SD*), Range	N (%)		
Age	37.2 (4.2), 29–46		34.5 (4.2), 23–47		10.8	0.001
Age of youngest child (in months)	10.2 (8.2), 0–36		11.2 (8.8), 0–36		0.5	0.462
Academic education ^a^		42 (89)		62 (89)	0.0	0.894
Above average income ^b^		36 (77)		46 (66)	1.6	0.208
Number of children	1.6 (1.0), 1–8		1.7 (1.0), 1–8		0.1	0.771
In a romantic relationship ^c^		67 (98.5)		98 (99)	FET	1.000
**Study Variables**	**Gay Fathers** **(N = 68)**		**Heterosexual Fathers** **(N = 99)**		**F**	
EPDS	6.1 (5.0), 0–21		4.7 (3.8), 0–18		4.1	0.044
SWLS	5.4 (1.0), 2.4–7.0		5.2 (0.9), 2.4–7.0		3.8	0.053
PTGI	3.9 (0.7), 1.5–5.5		3.5 (0.9), 1.1–5.8		8.9	0.003

^a^ Coded 0 = partial or full high school education and non-academic post-high school training, 1 = partial academic or academic education. ^b^ 0 = average income or less, 1 = above average income. ^c^ N = 68 for gay fathers and 99 for heterosexual fathers on this variable, Coded 0 = not in a romantic relationship, 1 = in a romantic relationship. EPDS = Edinburgh Postnatal Depression Scale; SWLS = Satisfaction with Life Scale; PTGI = Post-Traumatic Growth Inventory. FET = Fisher’s Exact Test.

**Table 2:** Regression analysis for testing the moderating effect of posttraumatic growth on group differences in postnatal depressive symptomatology.

Predictor	B	*t*	*p*
Group ^a^	8.1	2.5	0.014
PTGI	−0.2	−0.4	0.692
Group by PTGI	−1.7	−2.0	0.047

^a^ Heterosexual fathers (0) vs. gay fathers (1). Posttraumatic growth inventory; PTGI, Postnatal depressive symptomatology; EPDS.

**Table 3:** Simple slope analysis of the moderation effect of posttraumatic growth in the association between sexual orientation and postnatal depressive symptomatology.

Estimated at PTGI	Effect	*t*	*p*
Low level 2.8	3.3	3.1	0.002
Medium level 3.7	1.9	2.7	0.008
High level 4.6	0.4	0.4	0.684

Posttraumatic growth inventory; PTGI, Postnatal depressive symptomatology; EPDS. Effect indicates the mean difference in EPDS between gay and heterosexual fathers.

**Table 4:** Regression analysis for testing the moderating effect of satisfaction with life on group differences in postnatal depressive symptomatology.

Predictor	B	*t*	*p*
Group ^a^	12.0	5.3	<0.001
SWLS	−1.4	−3.3	0.001
Group by SWLS	−1.9	−3.0	0.003

^a^ Heterosexual fathers (0) vs. gay fathers (1) Satisfaction with life; SWLS, Postnatal depressive symptomatology; EPDS.

**Table 5:** Simple slope analysis of the moderation effect of satisfaction with life in the association between sexual orientation and postnatal depressive symptomatology.

Estimated at SWLS	Effect	*t*	*p*
Low level 4.3	3.9	4.6	<0.001
Medium level 5.3	2.1	3.6	<0.001
High level 6.2	0.3	0.4	0.664

Satisfaction with life; SWLS, Postnatal depressive symptomatology; EPDS Effect indicates the mean difference in EPDS between gay and heterosexual fathers.

## Data Availability

Data available on request from the authors.
